# Acute beetroot juice supplementation does not attenuate knee extensor exercise muscle fatigue in a healthy young population

**DOI:** 10.20463/jenb.2019.0008

**Published:** 2019-03-31

**Authors:** Seungyong Lee, Mark G. Abel, Travis Thomas, T. Brock Symons, James W. Yates

**Affiliations:** 1 Department of Pathology, Johns Hopkins University School of Medicine, Baltimore USA; 2 Department of Kinesiology and Health Promotion, University of Kentucky, Lexington USA; 3 College of Health Sciences, University of Kentucky, Lexington USA; 4 Department of Health & Sports Sciences, University of Louisville, Louisville USA

**Keywords:** Nitrate Supplementation, Muscle Fatigue, Muscle Efficiency, Blood Pressure

## Abstract

**[Purpose]:**

The effect of acute nitrate supplementation on muscle fatigue is largely unknown. This study aimed to evaluate the effect of acute nitrate supplementation on muscle fatigue.

**[Methods]:**

Thirty-five recreationally active subjects consumed 140 ml of beetroot (BR) juice (nitrate: 8 mmol·d^-1^) or placebo (PL) 12 and 2.5 hours before two exercise sessions. Peak torque was measured during 50 repetitions, at maximal effort, and during concentric knee extensions at 90°·s^-1^. Blood pressure (BP) was recorded pre- and post-exercise.

**[Results]:**

Peak torque, maximum work, rate of fatigue, and rate of work fatigue were similar between the BR and PL conditions. Post-exercise diastolic BP (BR: 67.2 ± 9.8 vs. PL: 64.5 ± 7.9 mmHg, *p* < 0.05) and mean arterial pressure (BR: 91.6 ± 9.3 vs. PL: 88.8 ± 8.2 mmHg, *p* < 0.05) were higher with BR supplementation.

**[Conclusion]:**

These findings suggest that the acute intake of BR juice had no effect on knee extensor muscle strength or fatigue but increased BP in a healthy recreationally active population.

## INTRODUCTION

Nitric oxide (NO) is synthesized from L-arginine by endothelial nitric oxide synthase (eNOS) and released by endothelial cells endogenously^1^^-^^3^. NO diffuses into the underlying smooth muscle of the blood vessel, activating the soluble guanylyl cyclase (sGC) pathway and evoking vasodilation^2^^,^^4^. A key regulator of NO release is the shear stress of blood over the endothelial cell lining^2^^,^^4^. Endogenous nitrates and nitrites are believed to be the stable inert end products of the NO oxidation mechanism (NO + O_2_ => Nitrite, Nitrite + HbO_2_ => Nitrate)^5^. However, a previous study^5^ suggested that the nitrite mechanism can be recycled to generate bioactive NO.

Dietary nitrate, found in foods such as green leafy vegetables, is one of the possible sources of exogenous NO^5^. Exogenous NO plays a pivotal role in many physiological responses to exercise, such as blood flow and blood pressure regulation, influencing the adenosine triphosphate (ATP) cost in muscle force generation, and enhancing oxidative phosphorylation^6^^-^^9^. Further, increasing dietary nitrates lowers the cost of oxygen consumption at submaximal workloads^10^^-^^20^. Oxygen consumption generally increases linearly relative to work rate^10^; thus, the cost of oxygen consumption is a predictable factor when people exercise at a given work rate.

Dietary nitrate supplementation enhances exercise economy and tolerance by increasing muscle contractile efficiency via decreasing total ATP utilization during muscle contractions and reducing muscle phosphocreatine utilization^10^. Subjects with a higher nitrate intake were found to have higher phosphocreatine levels than the placebo-treated group without altering pH levels after exercise. Moreover, the total ATP turnover rate was estimated to be lower with nitrate supplementation. Slower cross-bridge cycling may be accountable for the less ATP utilization with nitrate supplementation during muscle contractions^10^.

If cross-bridge cycling rate becomes slower by dietary nitrate supplementation with the force output held constant, it is reasonable to speculate that nitrate supplementation could change the rate of fatigue with less ATP depletion as measured during a muscle endurance task. However, the effects of nitrate supplementation on neuromuscular fatigue have been rarely investigated and remain controversial due to the duration of supplementation and various types of exercise^21^^,^^22^. Therefore, the primary purpose of the present study was to determine the effect of acute inorganic dietary nitrate intake, in the form of beetroot juice supplementation, on the rate of work fatigue during a muscle endurance task. Second, due to the documented vasodilatory effects of nitrate supplementation^1^^,^^4^^,^^9^^,^^19^^,^^23^, the present study sought to observe changes in hemodynamic outcomes before and after fatiguing exercise. It was hypothesized that acute nitrate supplementation would reduce the muscle fatigue rate during a knee-extensor muscle endurance task and would reduce systolic, diastolic, and mean arterial blood pressure before and after a fatiguing exercise.

## METHODS

### Experimental Approach to the Problem

This study was conducted as a double-blinded, randomized, crossover design to assess the effects of acute beetroot juice supplementation on muscle fatigue based on the use of dietary nitrate supplementation as an ergogenic aid for resistance training and athletic performance. Subjects completed two sessions of 50 repetitions of isokinetic knee extensor fatiguing exercise with the consumption of either beetroot (BR) juice or placebo (PL) beverage.

### Subjects

The procedures employed in this study were approved by the University of Kentucky Institutional Research Board. Thirty-five recreationally trained (i.e., exercise at least 2 d·wk-1) males and females (26 males, 9 females; Age: 23.6 ± 3.6 years; Height: 174.5 ± 10.2 cm; Body mass: 71.8 ± 13.3 kg; BMI: 23.4 ± 2.9 kg·m-2) were recruited for this study. The subjects were screened for participation using the Physical Activity Readiness Questionnaire (PAR-Q), food frequency questionnaire (FFQ), and a medical history questionnaire (MHQ). The FFQ inquired about subjects’ daily and weekly food consumptions to estimate daily nitrate intake. The PAR-Q and MHQ examined the subjects’ medical histories, physical characteristics, and weekly activity levels to verify that they met the inclusion criteria. The subjects were informed of the potential benefits and risks of the study before signing a consent form to participate. The subjects were excluded from the study if they had consumed more than 600 mg·d-1 of nitrate, reported knee problems, were unable to perform maximal effort knee extensions, were using antihypertensive medication, or had uncontrolled blood pressure.

### Procedures

The subjects were asked to report to the laboratory fully rested and were reminded to avoid strenuous exercise 24 hours prior to each testing period. In addition, each subject was given a list of foods with a high nitrate content and instructed to refrain from the consumption of these foods starting 48 hours prior to the first testing session and continued until all testing sessions were completed. Moreover, the subjects were encouraged not to use antibacterial mouthwash and chewing gum 48 hours before each testing session because these products kill oral bacteria that play a pivotal role in converting dietary nitrate to nitrite^18^. No instructions were given regarding caffeine consumption or smoking during participation in the experiment.

### Dietary Supplementation Procedures

#### Beetroot Juice 

Beet It stamina shot (James White Drinks, Ipswich, UK), a commercially available BR juice (~4.0 mmol of nitrate/bottle), was used as described elsewhere^21^^,^^22^^,^^24^. The contents of nitrate (0.3 g of dietary nitrate/bottle), calories (71 kcal/bottle), and protein (2.5 g/bottle) were found on the nutritional information label.

#### Placebo Beverage

The PL beverage was modified to be isocaloric and isonitrogenous compared to the BR beverage. Additional sugar (sugar syrup) and protein (Beneprotein powder, Nestle Nutrition, Vevey, Switzerland) were included to match the BR juice’s carbohydrate and protein composition and ensure that the nitrate content was the only difference between the two drinks. The PL beverage and BR juice had similar odor, color, taste, and appearances.

#### Intervention

Each subject completed identical testing procedures in the BR juice and PL conditions in two separated days, and the order of two tests were randomly assigned. The subjects ingested 70 ml × 2 bottles = 140 ml of either inorganic nitrate from BR juice or PL drink (black currant juice: negligible nitrate concentration). The nitrate concentration of each bottle of BR was ~4.0 mmol, and two bottles of BR (~8.0 mmol·d-1) in the current study showed comparable levels of nitrate used in a previous study (~5.5 mmol·d-1)^10^. The subjects consumed the first bottle of beverage 12 hours before the testing session and the second bottle of beverage 2.5 hours before the testing session. Both BR juice and PL beverage were randomly given to the subjects in identical black bottles by a blinded investigator who did not participate in data collection. The beverages were placed in a brown paper bag and given to the primary investigator to give to the subjects. A separate investigator kept track of the order of beverages and informed the principal investigator of the beverage identity after all testing was completed. All subjects had at least a 72-hour washout and recovery period between the two exercise testing sessions.

### Exercise Testing Procedure 

The knee extensor muscle group of each subject’s self-selected dominant leg was tested. Each subject completed exercise testing on three separate days, including one familiarization session and two testing sessions following the beverage consumption. Peak torque was measured on the Biodex dynamometer (Biodex System 3; Biodex, Shirley, NY, USA) by performing 50 maximal effort concentric knee extensions at 90°·s-1. This fatiguing exercise protocol for the extensor muscles was modified from the protocol used by Kawabata et al. (2000)^25^. All knee extensor testing was administered by the same investigator. Pre-testing was performed by each subject for them to become familiar with the device. The subject was seated, and the theoretical middle of the knee was visually aligned with the dynamometer’s axis of rotation. The position of the chair and the dynamometer were recorded so that they could be duplicated in subsequent testing sessions. During the test, the subjects were secured to the dynamometer by four different bands (two shoulders, one waist, and thigh straps) to limit upper-body and pelvic movements. This restriction helped isolate the testing muscle groups and minimized upper-body involvement that may confound torque generation. The total knee extension range of motion (ROM) was 80°. The ROM limits were set from 15° to 95° of flexion (0° = full leg extension). Prior to testing, the leg was weighed to correct the torque to include leg weight.

All subjects performed 50 maximal knee extensions at 90°·s-1 followed by passive knee flexion, also at 90°·s-1. Each subject was instructed prior to beginning the test to employ maximal effort by contracting the muscles as hard as possible for each repetition. Consistent verbal encouragement was provided during each test. The subjects were instructed to exhale during the knee extensor exercise. Peak torque and work for each knee extensor repetition were measured. Peak torque was determined as the single highest value attained from each repetition. In addition, since higher torque values were typically generated after the velocity exceeded 85°·s-1, isokinetic work was analyzed for 700 milliseconds and represented the area underneath the torque curve from the first point where the velocity surpassed 85°·s-1. The change in peak torque generation and work over the number of contractions was used as a fatigue index. Peak torque and work rate were averaged over five consecutive repetitions and expressed as a percentage. The 50 contractions were divided into 10 stages with five consecutive contractions each. The work rate from each repetition was measured in the same way as peak torque.

### Blood Pressure Measurement Procedures

Resting brachial blood pressure (BP) was manually taken after 10 minutes of quite sitting (i.e., pre-exercise BP). This BP was taken approximately 2.5 hours after the last BR supplementation since the last dose of BR juice was supplied 2.5 hours before each exercise test. The second BP (post-exercise BP) was taken immediately following (i.e., 10–20 s post-exercise) the knee extensor exercise. Mean arterial pressure (MAP) was calculated as [(2 × diastolic BP) + systolic BP] / 3.

### Statistical Analyses

Microsoft® Excel 2010 (Microsoft, Redmond, WA, USA) was used to manipulate the raw data. The raw data were manipulated into 50 contractions to identify the peak torque and work rate for each contraction. The peak torque and work per repetition across 50 contractions were separated by 10 repetition groupings of 5 repetitions each. The rate of fatigue was calculated as the fatigue index using the following equation:

Eq. 1 $$\text{Percent decrease=100-}\left[\left(\text{averaged last 5 repetitins/averaged first 5 repetitions}\right)\text{×100}\right]\text{.}$$

Peak torque and work rate were averaged over 5 consecutive contractions and then expressed as a percentage of the highest 5 contractions. Thus, the ratio of the peak torque and maximum work average in the first stage (the first 5 repetitions) to each of the subsequent 5 repetition groupings represented the relative fatigue rate (%). Paired sample t-tests were used to assess the differences between BR and PL in mean peak torque, mean maximum work, and BP outcomes. Changes in mean peak torque, maximum work rate across 50 maximal voluntary contractions, and the rate of work fatigue at multiple stages were analyzed by two-way ANOVA (supplement × time changes) repeated measures for BR and PL. The significance level was accepted at P values < 0.05. Data are expressed as mean ± standard deviation (M ± SD) unless stated otherwise. SPSS (version 20.0; SPSS, Chicago, IL, USA) was used to conduct the statistical analyses.

## RESULTS

### Mean Peak Torque and Mean Maximum Work Following Treatments

Table 1 illustrates the mean peak torque (Nm) and mean maximal work (J) during the 50 contractions across 35 subjects with BR and PL supplementation. There was no statistical difference between the BR and PL conditions in mean peak torque and maximal work across the 35 subjects.

**Table 1:** Comparison of peak torque and maximal work between beetroot (BR) and placebo (PL) beverage conditions for 35 subjects.

	BR	PL	*P* value	Effect size (Cohen's D)
Peak torque (Nm)	179.8 ± 46.7	180.6 ± 48.1	0.663	-0.074
Maximum work(J)	10,256 ± 2,845	10,230 ± 2,686	0.781	0.047

Values are mean ± SD.

Nm, Newton meter; J, Joule

### Changes in Peak Torque and Maximum Work Following Treatments

**Figure 1:**
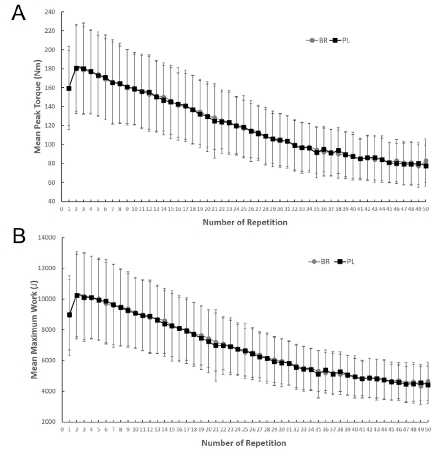
Changes in absolute mean peak torque generation (A) and maximum work rate (B) across 50 maximum voluntary contractions in 35 subjects in the beet root (BR) and placebo (PL) conditions. Values represent mean ± SD.

Changes in absolute peak torque and maximum work rate are shown in Figure 1. Both parameters showed a steady decline over the 50 contractions (changes in peak torque: *F* = 169.916, *p* < 0.0001; changes in maximum work: *F* = 279.713, *p* < 0.0001), but there was no difference (*p* > 0.05) between the PL and BR conditions.

### Changes in Muscle Fatigue Following Treatments

**Figure 2:**
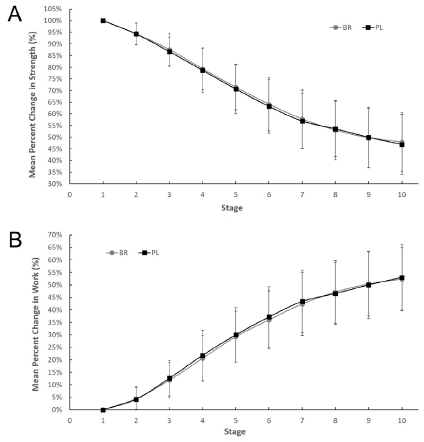
Percent changes in peak torque (A) and rate of work fatigue (B) over 10 stages in the beet root (BR) and placebo (PL) conditions in 35 subjects. Values represent mean ± SD.

There were no differences between BR and PL in the rate of fatigue by either peak torque or work over 50 contractions. Figure 2 shows the change in peak torque (Figure 2A) and maximal work (Figure 2B) over 10 stages for BR versus PL expressed as a percentage of mean remained strength. No treatment effect occurred indicating that BR supplementation had no effect (*p* > 0.05) on the rate of muscle fatigue by either change in peak torque or change in maximal work compared to PL treatment, while both conditions generated significant muscle fatigue (changes in peak torque: *F* = 471.414, *p* < 0.0001; changes in maximum work: *F* = 454.874, *p* < 0.0001) over 50 contractions.

### Changes in BP Following Treatments

The BP data are summarized in Table 2. No significant differences in resting BP were observed between the BR and PL conditions before fatiguing exercise. Post-exercise diastolic BP (DBP) and mean arterial pressure (MAP) were higher in BR supplementation than in PL supplementation, whereas there was no difference in post-exercise systolic BP (SBP).

**Table 2:** Blood pressure at pre- and post-exercise points with beetroot (BR) and placebo (PL) supplementation in 35 subjects.

Blood pressure (mmHg)	BR	PL	Effect size (Cohen's D)
Pre-systolic BP	111.7 ± 7.7	111.4 ± 6.8	0.047
Pre-diastolic BP	70.8 ± 8.7	70.1 ± 7.8	0.087
Pre-MAP	84.4 ± 7.8	83.8 ± 6.8	0.087
Post-systolic BP	140.3 ± 12.9	137.3 ± 15.1	0.265
Post-diastolic BP	67.2 ± 10.2^*^	64.5 ± 7.9	0.369
Post-MAP	91.5 ± 9.3^*^	88.8 ± 15.1	0.464

Values are mean ± SD. *p < 0.05 from placebo group as determined by paired-sample t-tests

BP, blood pressure; MAP, mean arterial pressure

## DISCUSSION

This investigation demonstrates that acute (only one day) nitrate supplementation in the form of BR juice does not alter the peak torque, maximum work rate, and the muscle fatigue rate (Table 1, Figure 2). Although resting BP was unaltered with BR juice supplementation (Table 2), elevated post-exercise DBP and MAP were observed (Table 2). These findings did not support the alternative hypotheses of the current study and were inconsistent with the improvement in performance and hemodynamics parameters observed by previous studies following dietary nitrate supplementation^10^^-^^20^. However, these findings are important as a first step in elucidating the effect of acute inorganic dietary nitrate on muscular fatigue and resistance exercise.

The current study demonstrated that BR supplementation had no positive effect on muscle force generation and muscular work. The data in this experiment coincide with the previous reports in the literature illustrating that 15 days of 500 ml BR juice supplementation had no effect on the force generation following 50 isometric maximum voluntary contractions in a healthy young population^26^. Seven days of nitrate supplementation also did not improve a series of maximum voluntary isometric contractions of the knee extensor muscles, while peak force was enhanced under involuntary contraction (i.e., low-frequency electrical stimulation)^27^. Similarly, muscle fatigue measured by the height and power of a countermovement vertical jump was not altered by one dose of BR juice^21^. However, the positive effects of acute periods of nitrate supplementation were previously demonstrated. For example, improvements in exercise capacity and exercise performance in cycling time trial performance^15^, decreased oxygen cost of moderate-intensity exercise^19^, and reduced peak oxygen consumption and a ratio of VO_2_ power at maximal intensity exercise^13^. Acute BR juice supplementation has also been conjectured to enhance physiological functions such as blood flow and BP regulation^9^, regulatory effects on the ATP cost of force production^7^^,^^8^, and cellular oxygen utilization and enhancement of oxidative phosphorylation efficiency^6^ in normal physiological state and during exercise, similar to after long-term ingestion of nitrate supplementation. These physiological responses following acute nitrate supplementation enable us to speculate a reduction in the rate of muscle fatigue. Nonetheless, the current study suggests that increased NO bioavailability following acute BR juice supplementation did not affect the fatigue rate compared to control.

This study showed that the rate of fatigue by measuring torque generation and work rate in each set of fatiguing exercise was similar in the BR and PL trials. Both trials (with BR and PL) proceeded approximately 52–53% of total fatigue at the end of 50 repetitions of isokinetic knee extensor exercise. The fatiguing exercise protocol used in this study was similar to that used in previous research^22^^,^^25^^,^^28^. Pincivero et al.^28^ quantified muscle fatigue by calculating the declining slope in peak torque, maximum work, and power from the single highest value from each contraction across 30 repetitions of maximal concentric knee extension and flexion contractions. The total fatigue rate produced in that study was about 30–40%^28^. Likewise, Kawabata et al.^25^ reported similar total fatigue outcomes (40–56%) when performing 50 repetitions of active flexion and extension using a faster angular velocity (180°·s-1) in a sample of power and endurance athletes (baseball players, soccer players, and marathon runners).

In contrast to the present study, it has been demonstrated that 6 days of BR juice supplementation increased muscle contractile efficiency in incremental knee extensor exercise by measuring muscle metabolic responses in low- and high-intensity exercise^10^. Bailey et al.^10^ suggested that dietary nitrate supplementation reduced ATP utilization in the muscle during exercise by decreasing muscle phosphocreatine degradation and ADP and phosphate accumulation and further increasing the cost of oxygen utilization in the muscle. However, their findings may have resulted from the peak work rate being established using a two-legged knee extension ergometer as opposed to the 50 maximal one-legged isokinetic contractions used in the present study. This is because peak oxygen uptake and peak work rate are not increased in proportion to muscle activity so that the one-legged knee extensor exercise resulted in higher peak oxygen uptake and peak work rate compared to one leg in the two-legged exercise^29^. Although muscle metabolic responses and oxygen consumption in one-legged isokinetic knee extension exercise after acute BR supplementation were not directly measured, the higher oxygen and ATP utilization during the one-legged knee extension exercise may result in a decreased cost of oxygen utilization in exercising muscle and increased ATP utilization.

The divergent findings between studies may be related to the different durations (one day of two doses vs. six days) of nitrate supplementation or dosage. Previous studies have administered BR juice (Beet It original) in 500 ml beverages which contained 5.1–6.2 mmol of nitrate·d-1^10^^,^^11^^,^^16^^,^^19^. All levels of nitrate-containing drinks resulted in significant increases in plasma nitrates and nitrites. Although this study did not measure the plasma nitrite level, we believe it reasonable to assume that the BR used in this study resulted in increases in plasma nitrates and nitrites. This study used acute doses of concentrated BR juice at 8 mmol·d-1 because it has been shown that acute doses of 5.2 to 6.2 mmol·d-1 nitrate supplementation resulted in a significant improvement of oxygen utilization efficiency and time-trial performance in elite cyclists^13^^,^^15^^,^^19^. Even though this study used a BR supplementation with higher nitrate concentration (8mmol·d-1) compared to the other studies (5.2 to 6.2mmol·d-1), we tested non-elite athletes and muscular endurance exercise under the mechanism of muscle contractile efficiency improvement instead of oxygen utilization efficiency in athletes which may have partly contributed to varying results. Nonetheless, a fatiguing exercise protocol such as used in the present study may require a longer duration of BR supplementation to produce decreases in the rate of fatigue. Vanhatalo et al.^19^ have shown that although both acute and chronic dietary nitrate supplementation lowers oxygen cost and BP, the peak power output and the work rate related to gas exchange threshold were higher after 15 days of BR ingestion.

In addition, previous studies used PL beverages made of nitrate-depleted BR^15^^,^^16^^,^^20^^,^^30^ or black currant juice with negligible nitrate content^10^^,^^11^^,^^19^^,^^31^ since the beneficial effect of BR supplementation is based on the systemic reduction of dietary nitrate to NO. The use of black currant juice and nitrate depleted BR allowed those studies to investigate the effect of BR as an ergogenic aid with nitrate acting as the bioactive component. However, it is unknown if other bioactive compounds in BR or PL beverage may play a role in decreasing the rate of fatigue^32^. The present study used blackcurrant juice as a PL beverage which contains the antioxidant vitamin C. Reactive oxygen species (ROS) which are increased by strenuous exercise and may play a significant role in fatigue generation in the working muscle because ROS elevates oxidative stress in exercising muscle^32^. High doses of pharmacologic antioxidants such as N-acetylcysteine have been shown to depress muscle fatigue in rodent muscles and undamaged human muscle^32^. Thus, we speculated that even though the testing beverages (BR, in this case) are supplied from natural food sources, antioxidant properties of both BR and PL may reduce oxidative stress in working muscle and further confound our outcomes related to muscle fatigue during and after exercise.

The BR juice contains other bioavailable antioxidants such as betalain, according to research conducted by Wootton and Ryan^33^. The authors suggested that BR supplementation, especially Beet it stamina shot, increases total antioxidant capacity in the post-digestion phase^33^. Again, antioxidants may play a role in lowering oxidative stress induced muscular fatigue by reducing ROS. In the current study, we approached the primary hypothesis that acute BR juice supplementation significantly reduces muscle fatigue, potentially due to the nitrate-induced improvement in muscle contractile efficiency. Even though the findings which indicated BR juice stamina shot contains anti-oxidants from the study^33^, there was no significant difference in the rate of muscle fatigue between BR and PL in the current study. Thus, one possible explanation is that anti-oxidant properties in the PL and BR juice might elicit similar outcomes on the rate of fatigue.

Similarly, our dietary control protocol and previous studies^10^^,^^11^^,^^19^^,^^31^ limited additional nitrate consumption other than BR prior to and during the study. Subjects were asked to refrain from consumption of foods rich in nitrates prior to and during the study. Thus, the combination of the compound from the other food sources, rather than dietary nitrate from BR juice alone, may not significantly affect the outcomes of this study. Other studies recorded foods and fluid that participants consumed 24 hours before the first test, and duplicated the foods and fluid in the subsequent trials to control dietary nitrate intake^15^^,^^16^. Even though Vanhatalo et al.^19^ allowed their subjects to have a normal diet which included vegetables with high nitrate and nitrite contents, plasma nitrite level was significantly increased after BR juice supplementation and cost of oxygen was reduced during submaximal exercise after BR juice ingestion^19^. Thus, this would suggest that the beneficial effect of BR supplementation on exercise efficiency is applicable without dietary control. The current study also instructed subjects not to use antibacterial mouthwash and chewing gum 48 hours before each testing session to avoid eliminating oral bacteria that play a pivotal role in converting dietary nitrate to nitrite^16^. This step ensures leading to an appreciable increase in plasma nitrites.

In the current investigation, resting BP did not differ between the BR and PL conditions. NO derived from dietary nitrate is a known vasodilator that can regulate the vasomotor activity of the systemic vasculature. Further, NO regulates BP and blood flow as well as tissue oxygenation at rest and after exercise^11^^,^^18^. Resting SBP decreases after various forms and durations (acute to 15 days) of nitrate supplementation including BR juice^9^^-^^11^^,^^15^^,^^16^^,^^19^^,^^20^^,^^23^, inorganic nitrate in a capsule^9^, and sodium nitrate^14^ supplementation. On the contrary, DBP and MAP were not changed with nitrate supplementation^9^^,^^10^^,^^15^. Similar to the current findings, neither SBP nor DBP is affected by nitrate supplementation in the resting state^18^^,^^30^^,^^31^.

Few previous studies measured post-exercise BP. At 2 minutes post-exercise, DBP was decreased by 8 mmHg following sodium nitrate supplementation^18^. In contrast, Bond et al. in 2012 reported that SBP and DBP were increased at cessation of exercise, at one-minute post-exercise and two-minutes post-exercise with six sets of maximal intensity rowing performances^31^. Although the DBP at one-minute post-exercise was not statistically significant (*p* = 0.056), it seems to align with the results of the current study. It can be speculated that upper-body muscle contraction involved in the isokinetic knee extensor exercise may be responsible for the acute effect on DBP and MAP in the upper body muscles. In 2015, Kim et al.^34^ demonstrated that acute BR juice supplementation did not improve brachial artery vasodilatory function, blood flow, or shear stress during the exercise and resting period. Although nitrate supplementation has been shown to have positive systemic effects, there was no direct effect on brachial artery vasodilation, which may be associated with unchanged SBP measured in the brachial artery at rest and during fatiguing exercise in the current study.

While the reason for the BP results in the current study is unknown, a spontaneous Valsalva maneuver during maximal voluntary contraction may additionally increase BP^35^. However, we asked the subjects to breathe normally during the knee extensor exercise to avoid this maneuver. We observed that most subjects (27 of 35) did not perform this maneuver, while some needed a verbal reminder during 50 contractions of isokinetic knee extensor exercise. In addition, the antioxidant contents of both BR and PL might contribute to the BP changes we observed after fatiguing exercise. In a meta-analysis, Juraschek et al.^36^ found that vitamin C supplementation reduced both SBP and DBP significantly because vitamin C increases NO production by elevating eNOS as a potential vasodilator^36^. However, the doses and duration of supplementation reported by Juraschek et al. (500 mg·d-1 and 8 weeks, respectively) greatly exceeded the doses from our study (approximately 21 mg·d-1)^36^.

The present study has some limitations. First, this study did not measure plasma nitrate and nitrite levels for either treatment. However, BR supplementation provided a dietary nitrate dose (8 mmol·d-1) that has been shown to increase nitrite levels in the circulation. Also, physical state such as body weight should be considered in future studies because the current study utilized the same concentration and dose of BR juice, which might influence the outcome. Second, we did not assess the major components of the PL beverage to determine if it possessed any vasodilation properties. Ideally, future studies need to monitor whether reactive oxygen species are increased following fatiguing exercise stimuli and the effect of antioxidants on ROS during fatiguing exercise. Third, each subject chose their own exercise testing time; thus, some of the subjects did not perform the exercises at the same time for each test. This may influence BP and performance by impacting diurnal variations in the vascular parameters.

The results of this study suggest that acute BR supplementation has no effect on the rate of muscular fatigue as measured by repeated knee extensor exercise. Acute BR supplementation before knee extensor exercise appears to have no positive effect on the maximal force generation or maximum work rate. In addition, comparable to several previous studies, the hemodynamic parameters did not change in the resting state after acute BR juice supplementation versus PL supplementation. In contrast, the present study showed increased DBP and MAP after fatiguing exercise. These outcomes suggest to practitioners, coaches, and athletes that acute BR juice supplementation may not have an ergogenic benefit on muscle fatigue, improve muscle efficiency in resistance exercise, or power performance. Although further research is guaranteed to prove the influence of BR on the hemodynamic alterations, acute BR juice supplementation may negatively affect post-exercise hemodynamics by increasing DBP and MAP following the fatiguing exercise.
